# Accounting for treatment by center interaction in sample size determinations and the use of surrogate outcomes in the pessary for the prevention of preterm birth trial: a simulation study

**DOI:** 10.1186/s13063-016-1433-y

**Published:** 2016-07-05

**Authors:** Andrew R. Willan

**Affiliations:** Ontario Child Health Support Unit, Sickkids Research Institute, 555 University Avenue, Toronto, ON M5G 1X8 Canada

**Keywords:** Treatment by center interaction, Sample size requirements, Bayesian decision models, Surrogate outcomes

## Abstract

**Background:**

The Pessary for the Prevention of Preterm Birth Study (PS3) is an international, multicenter, randomized clinical trial designed to examine the effectiveness of the Arabin pessary in preventing preterm birth in pregnant women with a short cervix. During the design of the study two methodological issues regarding power and sample size were raised. Since treatment in the Standard Arm will vary between centers, it is anticipated that so too will the probability of preterm birth in that arm. This will likely result in a treatment by center interaction, and the issue of how this will affect the sample size requirements was raised. The sample size requirements to examine the effect of the pessary on the baby’s clinical outcome was prohibitively high, so the second issue is how best to examine the effect on clinical outcome. The approaches taken to address these issues are presented.

**Results:**

Simulation and sensitivity analysis were used to address the sample size issue. The probability of preterm birth in the Standard Arm was assumed to vary between centers following a Beta distribution with a mean of 0.3 and a coefficient of variation of 0.3. To address the second issue a Bayesian decision model is proposed that combines the information regarding the between-treatment difference in the probability of preterm birth from PS3 with the data from the Multiple Courses of Antenatal Corticosteroids for Preterm Birth Study that relate preterm birth and perinatal mortality/morbidity. The approach provides a between-treatment comparison with respect to the probability of a bad clinical outcome. The performance of the approach was assessed using simulation and sensitivity analysis.

Accounting for a possible treatment by center interaction increased the sample size from 540 to 700 patients per arm for the base case. The sample size requirements increase with the coefficient of variation and decrease with the number of centers. Under the same assumptions used for determining the sample size requirements, the simulated mean probability that pessary reduces the risk of perinatal mortality/morbidity is 0.98. The simulated mean decreased with coefficient of variation and increased with the number of clinical sites.

**Conclusion:**

Employing simulation and sensitivity analysis is a useful approach for determining sample size requirements while accounting for the additional uncertainty due to a treatment by center interaction. Using a surrogate outcome in conjunction with a Bayesian decision model is an efficient way to compare important clinical outcomes in a randomized clinical trial in situations where the direct approach requires a prohibitively high sample size.

## Background

The Pessary for the Prevention of Preterm Birth Study (P3S) is an international, multicenter, randomized clinical trial (RCT) to be conducted in 80 clinical centers by the Centre for Mother, Infant, and Child Research at the Sunnybrook Research Institute in Toronto, and is designed to answer the following primary question: Does the introduction of the Arabin pessary reduce the probability of preterm birth in pregnant women with a short cervix? The Arabin pessary is a simple, non-invasive therapeutic option for preventing preterm birth. Closing the cervix with a silicone ring (that is removed at term gestation) is a simple, relatively painless procedure that can be performed in an office setting and does not require surgery, hospitalization or anesthesia. To be eligible for randomization, women must present with a singleton pregnancy between 14 and 24 weeks gestation, and be identified by ultrasound to have a cervical length of between 10 and 24 mm. Randomization will be by random-sized blocks, stratified by center and gestational age. The primary outcome is preterm birth (i.e., birth at or prior to 33^+6/7^ weeks’ gestation). Since there is no agreed-upon standard of care, women will be randomized between (1) the current management of short cervix as determined by the center’s policy or at the attending physician’s discretion (Standard) and (2) Arabin pessary together with any additional care at the attending physician’s discretion (Treatment).

Two issues regarding sample size and power arose in the design and proposed analysis of P3S. The investigators assume that since treatment in the Standard Arm will vary between clinical centers, so might the probability of preterm birth in that arm. However, since all patients in the Treatment Arm are allocated to pessary, the investigators assume that due to a floor effect, if treatment does reduce the probability of preterm birth, the additional effect of any other interventions will be minimal and the probability of preterm birth in the Treatment Arm will not vary substantially between centers. As a result the treatment effect will vary between centers. This will increase the uncertainty with which the overall treatment effect is estimated and require a larger sample size to maintain type I and II error probabilities. To account for this in the analysis a random-effects model that allows the treatment effect to vary by clinical center (i.e*,* treatment by center interaction) should be used. The above assumption for the Treatment Arm is not required for the analysis using a random-effects model to be valid, although relaxing it could increase the uncertainty further. A bigger issue, and the one addressed in this paper, is what effect will a treatment by center interaction have on the sample size requirements.

Many authors have discussed the issue of center effect in RCTs [1–13]. Investigators who suspect that the treatment effect varies by clinical center (i.e., the existence of treatment by center interaction) have three strategies to choose from. The first is to ignore the issue. The second is to adopt a model with a fixed effect for center. The third is to adopt a model with random effect for center. The disadvantage of the “ignore it” strategy is that if a treatment by center interaction does exist then the uncertainty regarding the estimated treatment effect will be underestimated, leading to an unknown inflation of the type I error probability. The disadvantage of a fixed-effects model is that the inference is restricted to the centers in the trial. As argued by Feaster et al. [13] and others, inference from a fixed-effects model is more appropriate for an early phase III trial with a small number of centers, the positive results of which should lead to a more pragmatic trial with more centers and employing a random-effects model. Furthermore, for a fixed-effects model the inference for each center is based solely on their own data since there is no parameter in the model that represents an overall treatment effect. On the other hand, the random-effects model does contain a parameter for overall treatment effect and adjusts the uncertainty to account for the between-center variance in treatment effect. In addition, shrinkage-type estimation, which uses the data from all centers, can be used to provide center-specific inference [14]. One must assume that the centers in the trial are a random sample of centers to which inference is to be made. Although this may seem a strong assumption, it is reasonable to expect that most RCTs are done to make inference to a population broader than just the centers in the trial, and without this assumption, valid inference beyond the centers in the trial is problematic. One disadvantage of the random-effects model is that it is generally accepted that a reasonably large number of centers are required to provide a robust estimate of the between-center variance [12]. In addition, since the between-center variance is usually unknown in advance, sample size determinations that account for it can be challenging. Raudenbush and Liu [8] provide power and sample size solutions adjusting for treatment by center interaction when the outcome variable is continuous. Less well developed are methods for adjusting sample size requirements for a binary outcome variable.

The second issue is how best to answer the question: Does the introduction of the pessary reduce the probability of a bad clinical outcome for the babies? The sample size to answer this question directly by observing the clinical outcome of the infants in P3S is prohibitively high. As an alternative the investigators propose a Bayesian approach, combining the information regarding the between-treatment difference in the probability of preterm birth from P3S with the data from the Multiple Courses of Antenatal Corticosteroids for Preterm Birth Study (MACS) [15] that relate preterm birth and perinatal mortality/morbidity. This analysis will provide an estimate of the reduction in the probability of the clinical outcome from the introduction of the pessary. The question of concern to the investigators is: Will the proposed analysis answer the question regarding clinical outcome with sufficient certainty?

Many authors [16–30] have discussed the issues related to using surrogate outcomes in RCTs. Ellenberg and Hamilton [16] discuss the use of a tumor marker as a surrogate for tumor response and emphasize the value of identifying valid surrogates for clinical outcomes that have insufficient sensitivity and specificity. Although Wittes, Lakatos and Probstfield [17] conclude that the use of valid surrogate outcomes can reduce sample size and shorten trial durations, they point out several problems that can arise, especially regarding informative censoring. A criterion proposed by Prentice [18], and supported by others [27, 28], for a valid surrogate is stated as follows: a variable for which a valid test of the null hypothesis of no treatment effect is also a valid test of the corresponding hypothesis based on the clinical outcome. The author discusses the implementation of the criterion and applies it to examples given in [16, 17, 21]. Freedman, Graubard and Schatzkin [19] take issue with the implementation of the Prentice criterion, arguing that unless the observed treatment effect exceeds its standard error by a factor of four, the procedure will usually only lead to a weak validation of the surrogate outcome. Fleming et al. [20] discuss the implementation of the Prentice criterion in the context of cancer and AIDS trials. Day and Duffy [22] demonstrate how, in the British Breast Screening Frequency Trial, the use of surrogate outcomes can dramatically reduce the variance of the estimate of treatment effect and shorten the duration of the trial. Fleming and DeMets [23] argue that the Prentice criterion for a valid surrogate is often not meet and use examples to support their argument. They point out that numerous pathways might affect the clinical outcome, not all which will be mediated through the surrogate. The authors also raise the issue that the interventions might affect the clinical outcome in ways other than through the surrogate. Daniels and Hughes [24] proposed a Bayesian meta-analysis approach for examining the association between the between-treatment difference in the surrogate and the between-treatment difference in the clinical outcome. Buyse and Molenberghs [25] take issue with the Prentice procedure for validating surrogate outcomes. They propose an alternative method for validation and provide an illustration for the situation where the surrogate and clinical outcome are either both normal or both binary. These methods are extended by Molenberghs, Geys and Buyse [29] for the situation where the surrogate is binary and the clinical outcome is continuous, and vice versa. Begg and Leung [26] argue that conceptual strategy for the Prentice procedure is flawed and propose an alternative structure for validating surrogate outcomes. Baker, Izmirlian and Kipnis [30] address the issues raised by Day and Duffy [22] and Begg and Leung [26].

The issue of sample size inflation is addressed in the next section where simulations are used to determine the required sample size in the presence of treatment by center interaction. In the section following that, a Bayesian model is proposed for comparing treatment arms with respect to perinatal mortality/morbidity by combining the information regarding the between-treatment arm difference in the probability of preterm birth from P3S with the information relating preterm birth and perinatal mortality/morbidity from MACS. The performance of the proposed analysis is assessed using simulations. Although the two issues are seemingly separate, they are related since both are associated with the same trial, and both deal with power and sample size questions. The first issue deals with loss of power due to between-center variation in treatment effect, while the second deals with lack of power because the clinical outcome is so rare.

## Methods

The P3S is currently under review for funding from the Canadian Institutes for Health Research. If funding is received then the trial will be registered and the appropriate ethics approval will be sought by the coordinating center and all the clinical centers. If P3S is funded then informed consent will be sought for each participant enrolled.

### Between center treatment-effect variation and sample size

Simulations were used to determine sample size under the assumption that the treatment effect, if it exists, will vary between centers. To model the assumption for the Standard Arm, the preterm probability was assumed to vary between centers following a Beta distribution, with parameters *a* and *b*. That is, π_*Si*_ ~ *Beta*(*a*, *b*), where π_*Si*_ is the preterm probability on the Standard Arm in the *i*^th^ clinical center. The Beta distribution was chosen because it yields values between 0 and 1, and as illustrated below, the values for *a* and *b* can be chosen to provide the desired mean and between-center variance. Furthermore, the Beta distribution is used in Bayesian analysis for proportions since it is conjugate to binomial sampling. That is, if the prior distribution for a proportion is Beta, then with binomial sampling, the posterior distribution will also be Beta. The parameters *a* and *b* were chosen to provide (1) a mean 0.3 that reflects the current preterm probability as determined by available evidence [15, 31] and (2) as the base case, a coefficient of variation (standard deviation/mean) of 0.3 to reflect a reasonable amount of between-center variation. In the results section solutions for values of the coefficient of variation ranging from 0.1 to 0.4 are given for comparison.

Since π_*Si*_ ~ *Beta*(*a*, *b*),

$$ E\left({\pi}_{Si}\right)=\frac{a}{a+b} $$ and $$ V\left({\pi}_{Si}\right)=\frac{ab}{{\left(a+b\right)}^2\left(a+b+1\right)}, $$

where *E* and *V* are the expectation and variance functions, respectively. Therefore, for P3S:

$$ E\left({\pi}_{Si}\right)=\frac{a}{a+b}=0.3 $$ and $$ CV\left({\pi}_{Si}\right)=\frac{\sqrt{V\left({\pi}_{Si}\right)}}{E\left({\pi}_{Si}\right)}=\sqrt{\frac{ab}{{\left(a+b\right)}^2\left(a+b+1\right)}}/\frac{a}{a+b}=0.3, $$

where *CV* is the coefficient of variation function. Solving these equations yields:

$$ a=\frac{1-{c}^2d}{c^2\left(1+d\right)}\kern1em \mathrm{and}\kern1em b=\frac{1-{c}^2d}{c^2d\left(1+d\right)};\kern1em \mathrm{where}\kern1em c=CV\left({\pi}_{Si}\right)\kern1em \mathrm{and}\kern1em d=\frac{E\left({\pi}_{Si}\right)}{1-E\left({\pi}_{Si}\right)}. $$

Thus, *a* = 7.478 and *b* = 17.45, and for these parameter values there is a 95 % probability that a center’s preterm probability lies between 0.16 and 0.46, representing a reasonably wide range. (This is the variation in the true probabilities, not the variation in the observed proportions, which also include sampling variation.) The preterm probability in the Treatment Arm, denoted *π*_*T*_, for which the investigators required sufficient power was set at 0.225 and fixed across clinical centers, yielding under the alternative hypothesis, a mean relative risk reduction of 0.25 and a mean number needed to treat of about 13. By assuming a *CV*(*π*_*Si*_) = 0.3, 79 % of the centers in the Standard Arm have a probability of preterm birth greater than 0.225.

The simulation procedure to determine the required sample size proceeded as follows.

- A chosen sample size of patients was allocated randomly between the 80 centers with equal probability among centers with the constraint that there had to be at least six patients in each center
- Because randomization is to be stratified-blocked by center, within each center, half the patients were assigned to the Standard Arm and half to the Treatment Arm
- Those assigned to the Treatment Arm had their outcome determined by a Bernoulli distribution with probability 0.225
- Those assigned to Standard in *i*^th^ center had their outcome determined by a Bernoulli distribution with a probability that was sampled from *Beta*(7.478, 17.45)
- The resulting data were analyzed using a random-effects model, allowing treatment effect to vary between centers, using risk difference as the measure of treatment effect
- The simulation was replicated 10,000 times, and the proportion of replicates in which statistical significance (two-sided, 5 % level) was achieved was recorded
- If the proportion of replicates which achieved statistical significance was less (greater) than 80 %, the sample size was increased (decreased) and the procedure was repeated

### Assessing the treatment effect on clinical outcome

The required sample size if perinatal mortality/morbidity was the primary outcome is 37,500 per arm. This was determined by simulation in the same way as the entries in Table 1 and is based on having an 80 % power to achieve statistical significance (two-sided, level 0.05) if the addition of the Arabin pessary reduced the probability of a bad outcome from 1 % to 0.75 %, a 25 % reduction and a number needed to treat of 400. Again, it was assumed that the probability of a bad outcome in the Standard Arm varied between centers following a Beta distribution with a coefficient of variation of 0.3 and a mean of 0.01 (i.e., *Beta*(9.9, 89.1). A total sample size of 75,000 is impossibly high, and the investigators have proposed a Bayesian analysis combining the information regarding the between-treatment difference in the probability of preterm birth from P3S with the data relating preterm birth and perinatal mortality/morbidity from the singleton pregnancies from MACS. In MACS perinatal mortality/morbidity was a composite measure of perinatal mortality or serious neonatal morbidity, which included respiratory distress syndrome, bronchopulmonary dysplasia, severe intraventricular hemorrhage, cystic periventricular leukomalacia and necrotizing enterocolitis. Among the 1463 singleton pregnancies in MACS, 530 were preterm, of which 166 experienced perinatal mortality/morbidity, and of the 933 who were not preterm 5 did. This yields an estimated probability of perinatal mortality/morbidity for preterm babies of 166/530 = 0.31 and an estimated probability of freedom from perinatal mortality/morbidity for term babies of 928/933 = 0.99.

**Table 1:** Required sample size per arm as a function of the coefficient of variation and the number of clinical centers

Coefficient of variation	Probability that π_*Si*_ >0.225	Number of clinical centers				
		20	40	60	80	100
0	1	610	580	560	540	520
0.1	0.99	660	600	580	560	530
0.2	0.90	880	700	650	600	560
0.3	0.79	1840	1000	800	700	640
0.4	0.71	50,000	1700	1200	900	800

In this analysis the parameter of interest is *Δ* = *p*_*T*_ − *p*_*S*_, where *p*_*T*_ and *p*_*S*_ are the probabilities of perinatal mortality/morbidity in babies whose mothers are randomized to the Treatment and Standard Arms, respectively. Thus, *Δ* is the between-treatment arm difference in the probability of perinatal mortality/morbidity.

The quantity *p*_*T*_ = *π*_*ptb*|*T*_ × *τ*_*pmm*|*ptb*_ + *π*_*tb*|*T*_ × *τ*_*pmm*|*tb*_, where:

- *π*_*ptb*|*T*_ is the probability of preterm birth for mothers randomized to the Treatment Arm
- *τ*_*pmm*|*ptb*_ is the probability of perinatal mortality/morbidity in a baby who is born preterm
- *π*_*tb*|*T*_ = 1 − *π*_*ptb*|*T*_ is the probability of term birth for mothers randomized to the Treatment Arm
- *τ*_*pmm*|*tb*_ is the probability of perinatal mortality/morbidity in a baby who is born at term

Similarly, *p*_*S*_ = *π*_*ptb*|*S*_ × *τ*_*pmm*|*ptb*_ + *π*_*tb*|*S*_ × *τ*_*pmm*|*tb*_, where:

- *π*_*ptb*|*S*_ is the probability of preterm birth for mothers randomized to the Standard Arm
- *π*_*tb*|*S*_ = 1 − *π*_*ptb*|*S*_ is the probability of term birth for mothers randomized to the Standard Arm

Substituting and simplifying, we get:

$$ \varDelta =\left({\pi}_{ptb\Big|T}-{\pi}_{ptb\Big|S}\right)\times \left({\tau}_{pmm\Big|ptb}-{\tau}_{pmm\Big|tb}\right). $$

Thus, if there is a one-to-one correspondence between preterm birth and the clinical outcome, i.e. *τ*_*pmm*|*ptb*_ = 1 and *τ*_*pmm*|*tb*_ = 0 then *Δ* = (*π*_*ptb*|*T*_ − *π*_*ptb*|*S*_), and inference regarding the clinical outcome can be based solely on preterm birth. Since the conditions *τ*_*pmm*|*ptb*_ = 1 and *τ*_*pmm*|*tb*_ = 0 will never hold, inference about *Δ* will be made by deriving a probability distribution for it from the posterior distributions for *τ*_*pmm*|*ptb*_ and *τ*_*pmm*|*tb*_ based on the data from MACS, and for (*π*_*ptb*|*T*_ − *π*_*ptb*|*S*_) based on the data from P3S. Assuming the uninformative prior *Beta*(1, 1) for *τ*_*pmm*|*ptb*_ and for *τ*_*pmm*|*tb*_, and recalling that the Beta distribution and binomial sampling are conjugate, the posterior distributions [32], given the data, are:

- *τ*_*pmm*|*ptb*_ ~ *Beta*(*y*_*ptb*_ + 1, *n*_*ptb*_ − *y*_*ptb*_ + 1), where from *MACS n*_*ptb*_ is number of preterm births of whom *y*_*ptb*_ experienced the clinical outcome. Recalling that *n*_*ptb*_ = 530 and *y*_*ptb*_ = 166, then *τ*_*pmm*|*ptb*_ ~ *Beta*(167,365)
- *τ*_*pmm*|*tb*_ ~ *Beta*(*y*_*tb*_ + 1, *n*_*tb*_ − *y*_*tb*_ + 1), where from *MACS n*_*tb*_ is number of term births of whom *y*_*tb*_ experienced the clinical outcome. Recalling that *n*_*tb*_ = 933 and *y*_*tb*_ = 5, then *τ*_*pmm*|*tb*_ ~ *Beta*(6,929)

The distribution for (*π*_*ptb*|*T*_ − *π*_*ptb*|*S*_) will come from a random-effects regression model for the outcome of preterm birth using the P3S data. The model will include a fixed effect for Treatment Arm and a random effect for center, allowing for the treatment effect to vary by center. By assuming an uninformative normal prior, the posterior distribution for (*π*_*ptb*|*T*_ − *π*_*ptb*|*S*_) will be *Normal*(*μ*, *ν*), where *μ* is the estimate of the coefficient for treatment group and *ν* is the associated variance. Using the above distributions, a distribution for *Δ* will be sampled. From the sampled distribution, the probability that *Δ* is less than 0 (i.e., the probability that pessary reduces the risk of the perinatal mortality/morbidity) will be determined. In addition, 95 % credible intervals for *Δ* will be determined. By using uninformative priors the inference regarding *Δ* will be conditional on using the data from P3S and MACS only.

The performance of the proposed analysis was examined using simulation as follows:

- The trial of 700 per arm was simulated 10,000 times using the same assumptions for deriving the sample size given above
- For each simulation:
  - o The mean and variance of the estimator of *π*_*ptb*|*T*_ − *π*_*ptb*|*S*_ were determined
  - o 10,000 samples were taken from a normal distribution with that mean and variance
  - o 10,000 samples were taken from the Beta distributions for *τ*_*pmm*|*ptb*_ and *τ*_*pmm*|*tb*_, as given above
  - o For each sample *Δ* was determined as (*π*_*ptb*|*T*_ − *π*_*ptb*|*S*_) × (*τ*_*pmm*|*ptb*_ − *τ*_*pmm*|*tb*_) from the sampled values
  - o The proportion of samples for which *Δ* was negative was recorded

## Results

### Between-center treatment-effect variation and sample size

For the base-case parameters listed in the next sentence the required sample size is 700 patients per arm. *E*(*π*_*Si*_) = 0.3; *CV* = 0.3; *π*_*T*_ = 0.225; number of centers = 80; type I error probability = 0.05 (two-sided); and type II error probability = 0.2. To examine robustness with respect to the *CV* and number of centers, the associated sample size requirements are given in Table 1. The sample size required increases with the coefficient of variation, since larger *CV*s result in greater uncertainty, and decreases with the number of centers, as noted by Feaster et al. [13] and Raudenbush and Liu [8]. The effect of the number of centers is explained in the Appendix. For 80 centers and a *CV* of 0, the required sample size was 540 per arm. Therefore, the increase in total required sample size to account for the between-centers variance is 320.

For this example the assumption has been made that there is no between-center variation in the probability of preterm birth in the Treatment Arm. This assumption was made on the assessment that if the pessary does decrease the probability of preterm birth by 0.075 (i.e., the alternative hypothesis is true), a floor effect is expected, meaning that any additional interventions are unlikely to decrease the probability much further, and so even if the additional interventions vary between center, it is unlikely to result in between-center variation in the probability in preterm birth. To examine the robustness of the assumption in this example consider the following.

From the Appendix, under the assumption that each center randomizes *n* subjects, the variance of the estimated treatment effect is given by $V=\frac{2\left({\sigma}_S^2+{\sigma}_T^2\right)}{N}+\frac{v_S+{v}_T}{K}$,

where *K* is the number of centers;

*N* is the total sample size, *i.e., N* = *n × K*;

*σ*_*S*_^2^ = *π*_*S*_(1 − *π*_*S*_) is the between-patient variance in the Standard Arm;

*σ*_*T*_^2^ = *π*_*T*_(1 − *π*_*T*_) is the between-patient variance in the Treatment Arm;

*v*_*S*_ and *v*_*T*_ are the between-center variances in the Standard and Treatment Arms, respectively, where *v*_*i*_ = (*p*_*i*_ × *CV*_*i*_)^2^, for *i* = *S*, *T*.

Under the alternative hypothesis, *π*_*S*_ = 0.3 and *π*_*T*_ = 0.225. In this example *K* = 80 and *N* = 1400. Thus, if there is no between-center variation in either arm (i.e., *CV*_*S*_ = *CV*_*T*_ = 0, and therefore *v*_*S*_ = *v*_*T*_ = 0), then *V* = 5.49 × 10^− 4^. Based on our assumption that there is between-center variation in the Standard Arm but not the Treatment Arm (i.e., *CV*_*S*_ = 0.3 and *CV*_*T*_ = 0), *V* = 6.50 × 10^− 4^, representing a 18.4 % increase. Now if we allow some residual between-center variation in the Treatment Arm, say *CV*_*T*_ = 0.1, then *V* = 6.57 × 10^− 4^, which is a mere 0.97 % increase. The small effect of the between-center variability in the Treatment Arm relates to the fact that the between-center variance, *v*_*T*_, is the square of the product of *π*_*T*_ and *CV*_*T*_, both of which are smaller than the corresponding quantities in the Standard Arm. Even a *CV*_*T*_ of 0.15 increases the variance by only 2.19 %. The conclusion is that in this example the assumption of no between-center variation is reasonably robust, especially considering that 0.3 was selected as an upper bound for *CV*_*S*_.

### Assessing the treatment effect on clinical outcome

The average (over the 10,000 simulated trials) of the proportion of negative *Δ’*s was 0.98 and the 5th percentile was 0.87. This provides strong evidence that if the pessary reduces the probability of preterm birth from 0.3 to 0.225, and 700 patients per arm are recruited, there will be a very high probability of concluding that the pessary reduces the probability of perinatal mortality/morbidity. The 5th percentile and average proportion of negative *Δ’*s are given in Table 2 as a function of the number of centers and the between-center coefficient of variation of the probability of preterm birth in the Standard Arm. As expected the proportion of negative *Δ’*s increases with increasing number of clinical sites and decreasing coefficient of variation. To save computation time the values given in Table 2, except those of the base case, are based on 1000 simulated trials and 10,000 samples.

**Table 2:** The average and (5th percentile) of the proportion of negative *Δ’*s as a function of the coefficient of variation and the number of clinical centers

Coefficient of variation	Probability that π_*Si*_ > 0.225	Number of clinical centers				
		20	40	60	80	100
0	1	0.98 (0.88)	0.99 (0.94)	0.98 (0.93)	0.99 (0.93)	0.99 (0.94)
0.1	0.99	0.98 (0.87)	0.98 (0.92)	0.99 (0.92)	0.98 (0.92)	0.99 (0.94)
0.2	0.90	0.97 (0.88)	0.97 (0.85)	0.98 (0.90)	0.98 (0.90)	0.99 (0.92)
0.3	0.79	0.97 (0.87)	0.96 (0.79)	0.97 (0.85)	0.98 (0.87)	0.98 (0.90)
0.4	0.71	0.97 (0.86)	0.94 (0.73)	0.96 (0.79)	0.97 (0.85)	0.98 (0.89)

The SAS programs used to generate Tables 1 and 2 can be found at www.andywillan.com/downloads.

## Discussion

In a multicenter RCT a treatment by center interaction may exist for any number of reasons. It can exist because of differences in patient referral patterns, clinician skills, supportive care, implementation of the Treatment Arm, clinical evaluation or, as in P3S, with variations in the Standard Arm. The risk of a treatment by center interaction is often ignored [13]. This can lead to inflated type I error probabilities due to underestimating uncertainty, and inflated type II error probabilities due to inadequate sample sizes. It is well known that random-effect models provide valid estimates of variances of the estimated treatment effects, and the models can be adopted after a trial is completed if a treatment by center interaction is observed. However, sample sizes cannot be adjusted if an interaction is observed once the trial has been completed. In which case the inflation of type II error probability (i.e., reduction in power) has to be accepted. Modeling the effect that a treatment by center interaction has on sample size is challenging since it depends on many factors, many of which might be unknown at the time of trial planning. These include the number of centers, the degree to which the treatment effect varies between centers and the distribution of patients between centers. Other factors include the nature of the outcome variable and the metric used for the treatment comparison. Employing simulation and sensitivity analysis, as illustrated on the P3S above, is a fruitful, albeit time-consuming, approach. The simulation process depends on the specific causes of the between-center variability and other factors as discussed above. Consequently, many applications require a custom programming code.

The Bayesian decision model approach proposed in the previous section uses a surrogate outcome from an RCT in combination with data from other sources to make treatment comparison with respect to more clinical outcomes. To meet the Prentice criterion for a valid surrogate it must be on the causal pathway between the intervention (Treatment versus Standard) and the clinical outcome, and the entire effect of the intervention on the clinical outcome must be through its effect on the surrogate. Investigators are well advised to consider these issues carefully. Regarding P3S, it is firmly accepted that preterm birth is a risk factor for bad clinical outcome and that interventions that reduce the probability of preterm birth will reduce the risk such an outcome [33–35].

The use of a surrogate outcome in a Bayesian decision model is particularly useful when the clinical outcomes are rare or occur well in to the future. For binary outcomes the required sample size is dominated by the one over the square of the risk difference; the smaller the risk difference, the greater the required sample size. Assuming constant relative risks, the ratio of the risk difference for a surrogate to that for the clinical outcome equals the ratio of the corresponding risks of the outcomes in the Standard Arm. For example, in the P3S trial the risks in the Standard Arm for surrogate and clinical outcome are 0.3 and 0.01, respectively, and consequently one over the square of the risk difference for the clinical outcome is 900 times that for the surrogate. Even when the surrogate and clinical risks do not differ greatly, the decision model approach has distinct advantages for clinical outcomes that occur much later in time, such as survival. If long-term survival is the clinical outcome of interest, it might be the case that the research question will no longer be relevant after the required number of events have been observed since interest may have shifted to new interventions.

The requirement that the entire effect of the intervention (Treatment versus Standard) on the clinical outcome must be through its effect on the surrogate can be easily overlooked. For example, if relapse is used as a surrogate for survival, care must be taken that treatment does not negatively affect survival through some long-term adverse outcome. The other limiting factors are the relevance and strength of the evidence relating the surrogate to the clinical outcome. Critical appraisal of the evidence, including appropriateness of the sample and the methodology used is crucial for establishing the credibility of any application of a decision model.

The proposed Bayesian approach mirrors the decision analytic approaches used in health economics for assessing the cost-effectiveness of health care interventions [36]. A number of issues come to the fore concerning the quality of such analyses [37], and investigators must be prepared to defend the assumptions made in building the model. For P3S the model itself was very simple. Nonetheless, issues regarding the appropriateness and exhaustiveness of the “other sources” need to be addressed. These issues aside, the approach provides a very efficient means of addressing important clinical questions. For example, the sample size for P3S to compare Treatment Arms with respect to the clinical outcome total 75,000, more than 50 times the planned recruitment of 1400.

## Conclusion

Simulation techniques were used to address two issues relating to sample size and power for the pessary trial. Modeling the effect that a treatment by center interaction has on sample size is challenging because it depends on many factors, most of which might be unknown at the time of trial planning. Nonetheless, as illustrated above employing simulation and sensitivity analysis is a useful approach for determining sample size requirements while accounting for the additional uncertainty due to a treatment by center interaction.

It is often the case that an important clinical outcome is very rare or occurs many years after randomization. Using a surrogate outcome in conjunction with a Bayesian decision model is an efficient way to compare important clinical outcomes in a randomized clinical trial in situations where the direct approach requires a prohibitively high sample size or impossibly long follow-up. For particular examples simulations can be used to determine the power properties of employing such an approach.

## Abbreviations

CV, coefficient of variation; MACS, The Multiple Courses of Antenatal Corticosteroids for Preterm Birth Study; P3S, The Pessary for the Prevention of Preterm Birth Study; RCT, randomized clinical trial

## Appendix

Consider analyzing the data from a multicenter RCT as one might conduct a random effects meta-analysis. Let *V*_*i*_ be the variance of the estimator of the parameter of interest in center *i*. Then:

$$ {V}_i=\frac{\sigma_S^2}{n_{Si}}+{v}_S^2+\frac{\sigma_T^2}{n_{Ti}}+{v}_T^2, $$

where *σ*_*S*_^2^ and *σ*_*T*_^2^ are the between-patient variances of an observation and *n*_*Si*_ and *n*_*Si*_ are the sample size for center *i*, for Standard and Treatment Arms respectively, and *v*_*S*_^2^ and *v*_*T*_^2^ are the between-center variances of the parameter of interest for the Standard and Treatment Arms, respectively. For the most part *v*_*T*_^2^ has assumed to be 0, but for generalizability values other than 0 values are considered here.

For the sake of illustration assume that *n*_*Si*_ = *n*_*Si*_ = *n* for all *i*. Then:

$$ {V}_i=\frac{\sigma_S^2+{\sigma}_T^2}{n}+{v}_S^2+{v}_T^2=\frac{\sigma_S^2+{\sigma}_T^2}{N/(2K)}+{v}_S^2+{v}_T^2=\frac{2K\left({\sigma}_S^2+{\sigma}_T^2\right)}{N}+{v}_S^2+{v}_T^2, $$

where *N* is the total sample size and *K* is the number of centers. The variance of the overall estimate of the parameter of interest is the inverse of the sum over the *K* clinical sites of *V*_*i*_^− 1^. This can be shown to equal:

$$ \frac{2\left({\sigma}_S^2+{\sigma}_T^2\right)}{N}+\frac{v_S^2+{v}_T^2}{K}. $$

Thus, the variance of the overall estimate of treatment effect decreases as the number of centers increases, holding the total sample size fixed.
